# A Loop That Matters—An Unusual Case of Bow Hunter’s Syndrome

**DOI:** 10.3390/brainsci12050657

**Published:** 2022-05-17

**Authors:** Bartosz Gajewski, Ludomir Stefańczyk, Jacek J. Rożniecki, Mariusz Stasiołek, Małgorzata Siger

**Affiliations:** 1 Department of Neurology, Medical University of Lodz, 90-153 Lodz, Poland; bartosz.gajewski@stud.umed.lodz.pl (B.G.); jacek.rozniecki@umed.lodz.pl (J.J.R.); mariusz.stasiolek@umed.lodz.pl (M.S.); 2 Department of Radiology, Medical University of Lodz, 90-153 Lodz, Poland; ludomir.stefanczyk@umed.lodz.pl

**Keywords:** Bow Hunter’s syndrome, rotational vertebral artery occlusion, vertebral artery coiling

## Abstract

Bow Hunter’s syndrome (BHS), also known as rotational vertebral artery occlusion (VAO), is a rare entity in which vertebral artery is reversibly compressed due to rotation or extension of the head, causing vertebrobasilar insufficiency. Because of VAO, BHS should be considered as a possible life-threatening condition. Diverse aetiologies of BHS may trigger a broad spectrum of non-specific symptoms and may result in frequent misdiagnosis of this disorder in daily clinical practice. Herein, we present a case of BHS caused by previously non-described vascular aetiology.

## 1. Introduction

Bow Hunter’s syndrome (BHS), also known as rotational vertebral artery occlusion (VAO), is a rare condition in which vertebral artery is reversibly compressed due to rotation or extension of the head [1,2]. The most common aetiologies of BHS are atherosclerosis, thromboembolism, trauma, tumors, osteophytes, fibrous bands, chiropractic manipulation, surgery, sports accidents, atlantoaxial instability, intervertebral disc herniation, congenital anomalies of vertebrae, neck muscles hypertrophy and vascular malformation [1,3,4]. Vertebrobasilar insufficiency in BHS patients causes different clinical manifestations such as: syncope, vertigo or dizziness, nausea and/or vomiting, balance, gait and/or coordination disturbances, dysarthria, motor or sensory deficits, visual impairment and diplopia [1,3,4]. Moreover, recently, an interesting case of BHS due to compression of non-dominant vertebral artery with recurrent posterior inferior cerebellar artery (PICA) syndrome was described [5]. Due to non-specific symptoms, patients with BHS are usually misdiagnosed in daily clinical practice. The consequences of VAO may be life-threatening, therefore it is important to remind physicians about this syndrome, its clinical presentation and diagnostic algorithm.

Herein, we describe a case of BHS caused by previously non-described vascular aetiology.

## 2. Case Presentation

A 62-year-old male with medical history of arterial hypertension was admitted to the Department of Neurology because of recurrent episodes of syncope induced by left neck rotation, in 3 preceding months. The first episode occurred while the patient had been driving a car. Additionally, 5 months prior to hospitalisation, the patient had transient incidents of dizziness and blurred vision associated with left neck turn. The patient had a history of smoking, he denied trauma or other concomitant diseases. In physical and neurological examination, no abnormalities were found apart from the observed near-syncopal event during submaximal left neck rotation. During this maneuver, the patient perceived a crescendo sound in the head. He took a “defensive position” of the neck, in order to eliminate and then avoid such symptoms.

Computed Tomography (CT) of the head was normal (Figure S1a). These findings were confirmed in Magnetic Resonance Imaging (MRI) of the brain which showed only a few, small, non-specific, non-contrast enhancing hyperintense lesions on fluid attenuated inversion recovery (FLAIR) and T2-weighted (T2W) images in the periventricular white matter of both hemispheres (Figure S1b,c).

CT of the cervical spine showed multilevel, cervical spondylosis with osteophytosis of the vertebral bodies and irregularities of the vertebral endplates (Figure S2a). MRI of the cervical spine revealed cervical spondylosis with spinal canal stenosis (Figure S2b). To exclude external compression of VAs, CT and MRI of the cervical spine was assessed by an experienced radiologist. Chest radiogram did not show the presence of accessory ribs.

**Figure 1:**
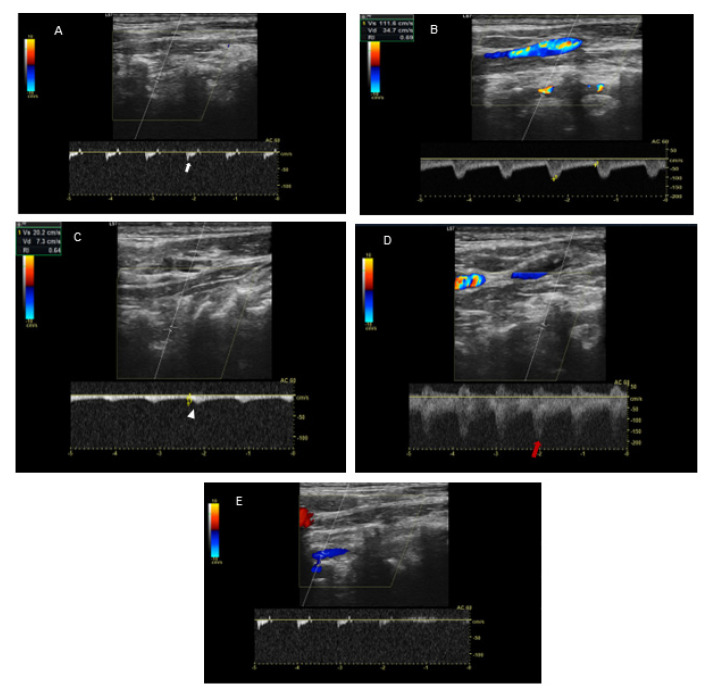
USGD in the neutral, left and right-side head rotations. Panel (**A**): USGD in the neutral position of the head demonstrates R-VA hypoplasia (width: 1.8 mm; N < 2–3 mm) with an increased blood flow resistance (PSV/EDV = 28/0 cm/s; RI = 1) (*white arrow*). Panel (**B**): USGD in the neutral position of the head demonstrates normal flow velocity in L-VA. Panel (**C**): USGD in left-side head rotation demonstrates post-stenotic flow velocity decline in L-VA (from 111/35 cm/s in neutral position to 20/7 cm/s in rotation; RI drop from 0.69 to 0.64)—the tardus and parvus wave (*white arrowhead*). Panel (**D**): USGD in left-side head rotation demonstrates a turbulent flow in L-VA with increased up to 200 cm/s peek systolic velocity at C4 level (*red arrow*). Panel (**E**): USGD in right-side head rotation demonstrates no changes in the blood flow.

Doppler ultrasonography (USGD) of cervical vessels was performed in the neutral position and in left-side rotation of the head. In the neutral position of the head, USGD detected right vertebral artery (R-VA) hypoplasia (width: 1.8 mm; N < 2–3 mm) [6] with an increased blood flow resistance (PSV/EDV = 28/0 cm/s; RI = 1) (Figure 1A). Flow velocity in L-VA in this position was normal (Figure 1B). Moreover, in the internal carotid arteries (ICA), atheromatous plaques up to 1.7 mm thick were detected, yet no significant stenosis was found. During left-side rotation of the head, flow velocity in L-VA significantly declined (from 111/35 cm/s to 20/7 cm/s; RI dropped from 0.69 to 0.64) (Figure 1C) behind a certain point (at C4 level), where a turbulent flow with increased up to 200 cm/s peek systolic velocity was observed (Figure 1D. No flow abnormalities in R-VA were revealed during right-side rotation (Figure 1E).

**Figure 2:**
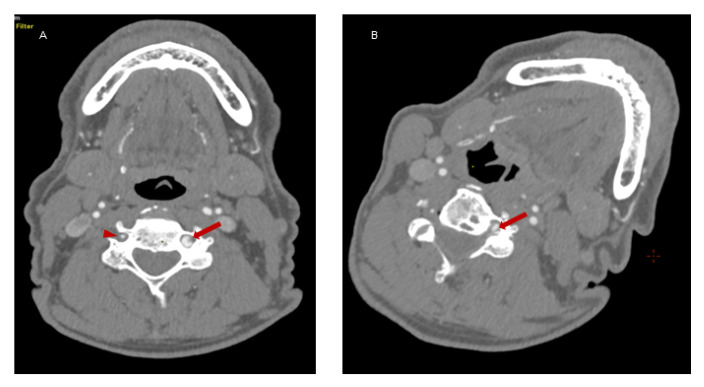
Dynamic CT angiography in the neutral head position and left-side rotation. Panel (**A**): Axial D-CTA in the neutral head position demonstrates L-VA loop in V2 segment (*red arrow*) at C4 level and R-VA hypoplasia (*red arrowhead*). No L-VA stenosis in this position. Panel (**B**): Axial D-CTA in left-side head rotation demonstrates the narrowing of L-VA lumen (up to 2 mm × 3.2 mm) that causes a dynamic 60% L-VA stenosis in the loop at C4 level (*red arrow*).

**Figure 3:**
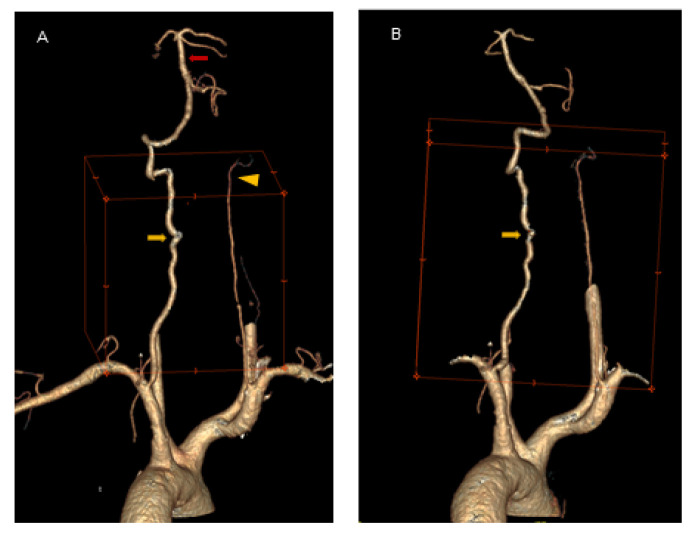
3D reconstruction in the neutral head position and left-side head rotation. Panel (**A**): Coronal 3D reconstruction of the vertebral arteries in the neutral position of the head demonstrates L-VA loop in V2 segment at C4 level (*yellow arrow*) and R-VA hypoplasia (*yellow arrowhead*). No L-VA stenosis in this position. BA is formed exclusively by L-VA (*red arrow*). Panel (**B**): Coronal 3D reconstruction of the vertebral arteries in left-side head rotation demonstrates the narrowing of L-VA lumen (up to 2 mm × 3.2 mm) that causes a dynamic 60% L-VA stenosis in the loop at C4 level (*yellow arrow*).

Because of the suspected dynamic stenosis, a dynamic CT angiography (D-CTA) of the carotid, vertebral and cerebral arteries was performed. Firstly, the imaging was conducted with the patient’s head in the neutral position. It revealed atheromatous plaques in both carotid bulbs (slightly calcified; maximum thickness: 2.8 mm) with no hemodynamically significant stenosis, grade 2 of kinking of R-ICA and a tortuosity of L-ICA. Furthermore, hypoplasia of R-VA was demonstrated (widths: R-VA 1.9 mm; L-VA 3.6 mm). L-VA had a tortuous course and it formed a loop at the level of the C4 vertebrae. There was no L-VA stenosis in this position (Figure 2A). Subsequently, D-CTA was acquired during left-side rotation of the head. In this examination the lumen of L-VA was narrowed up to 2 × 3.2 mm, causing 60% stenosis according to the NASCET criteria (Figure 2B) [7]. 3D reconstructions of this pathology were also obtained (Figure 3A,B). It was also observed that basilar artery (BA) was formed exclusively by L-VA. No abnormalities of other cerebral arteries were observed.

No significant changes in the heart rhythm were detected in twenty-four hours continuous electrocardiographic monitoring (Holter ECG). Transthoracic echocardiography (TTE) revealed decreased regional contractility of the left ventricle’s wall and a normal ejection fraction (EF = 52%; N ≥ 50%) [8].

The patient was evaluated by a vascular surgeon and disqualified from any surgical procedure. Consequently, a conservative treatment with acetylsalicylic acid and statin was implemented in order to prevent ischaemic stroke and treat hyperlipidaemia. The patient was instructed to avoid the position which causes the symptoms and referred to physiotherapy outpatients clinic. A rigid cervical collar was recommended. Regular neurological and radiological control examinations were also recommended. In the period of 3 months till the follow-up evaluation, the patient did not experience any new neurological symptoms.

## 3. Discussion

BHS was firstly described by Sorensen [1]. Its prevalence is not exactly known but considered to be slightly higher in men, at the fifth to the seventh decade of life [2,4]. There is a broad spectrum of BHS aetiologies and in some patients the cause cannot be specified [2]. BHS may be a harmful condition resulting in the insufficiency of the posterior circulation, triggering symptoms of stroke in the brainstem and the cerebellum. Even though most of the clinical symptoms of our patient are regarded as typical for BHS, tinnitus in the course of BHS episodes has been found rarely [5].

Digital Dynamic Subtraction Angiography (DDSA) remains the gold standard in the diagnostic process, however this invasive method requires the application of extensive radiation and restriction of patient’s position during the examination. Therefore dynamic USGD with the following D-CTA are recommended as less invasive tools in determining the diagnosis of BHS [2,3,4]. Management of BHS includes conservative treatment or interventional approaches—open surgery or endovascular procedure [2,3,4]. The majority (52%) of BHS cases are driven by pathologies located below C2 vertebrae. The most common anatomic location of VA occlusion is at C3-C7 vertebrae level [2], as it was also detected in our patient. Among possible pathomechanisms of BHS, vascular pathology is regarded as one of the least frequent. It was reported only in 11 cases, which equals 5% of the documented BHS patients [2]. Only a few cases of patients with isolated VA hypoplasia as the leading cause of BHS have been published [9,10,11,12]. There was also a case of BHS caused by tortuosity of L-VA in V1 segment with contralateral VA hypoplasia [13]. Dissection of VA was reported to be the cause of BHS in another case [14]. One publication illustrated an atherosclerotic stenosis of VA combined with spondylosis responsible for VAO [15]. An arterial embolic aetiology was demonstrated in one study [16]. In yet another publication, the described subject developed BHS due to a compression of persistent first intersegmental artery, a rare variant of VA, under an incomplete arch of C1 vertebrae [17]. BHS secondary to pseudoaneurysm of V3 segment with contralateral VA hypoplasia [18] and a spasm of VA with an aneurysm in its further course [1] were also described. It is also very important to highlight that anatomical variants can result in atypical BHS. In the case reported by Di Stefano et al. [5] unique symptoms from PICA were caused by unusual compression of the non-dominant VA at the level of the entrance in the inter-transversal canal.

Unlike the aforementioned examples, our case brings light to a novel, previously non-described vascular aetiology of BHS, which is the coiling of L-VA in V2 segment combined with contralateral VA hypoplasia. Most importantly, R-VA hypoplasia is the reason for the lack of the blood flow compensatory mechanism, resulting in transient ischaemia of the posterior cranial fossa structures.

## 4. Conclusions

BHS is a rare disorder which should be taken into consideration in differential diagnosis of neurological symptoms without obvious aetiology. D-CTA is recommended as the most accurate and applicable diagnostic method. Low prevalence of BHS results in its frequent misdiagnosis. It might be unacknowledged by physicians in daily clinical practice. Therefore, it is important to point out that in some cases it is a potentially curable disorder and should be considered in the differential diagnostic process of TIA or ischaemic stroke, especially in young patients.

## Data Availability

All clinical information is available in the patient’s medical record in the Department of Neurology, Medical University of Lodz. Radiological examinations are available on the local server in the Department of Radiology, Medical University of Lodz.

## Supplementary Materials

The following supporting information can be downloaded at: https://www.mdpi.com/article/10.3390/brainsci12050657/s1, Figure S1: Head CT and brain MRI, Figure S2: CT and MRI of cervical spinal cord, Patient Consent Form.
