# Supplementary material for: A Loop That Matters—An Unusual Case of Bow Hunter’s Syndrome
Source: Brain Sci. 2022 May 17;12(5):657. doi: 10.3390/brainsci12050657 (PMC9139719; doi:10.3390/brainsci12050657)
Supplement: Supplementary file 1 [file brainsci-12-00657-s001.zip › brainsci-1729335-supplementary.pdf]

Figure S1. Head CT and brain MRI

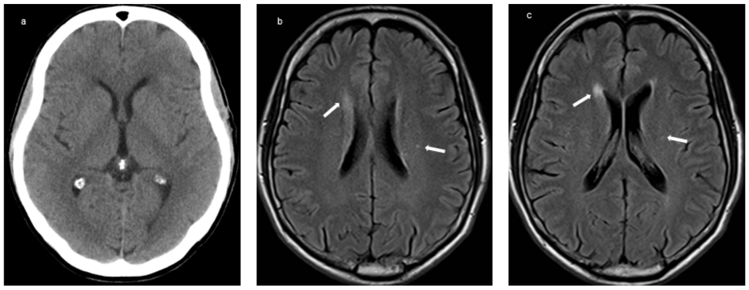

Figure **S1a:** Normal head CT, Figure **S1b, S1c.** Brain MRI demonstrates a few, small, non-specific, non-contrast enhancing hiperintense lesions on fluid attenuated inversion recovery (FLAIR) and T2-weighted (T2W) images in the periventricular white matter of both hemispheres (*white arrows).*

Figure S2. CT and MRI of cervical spinal cord

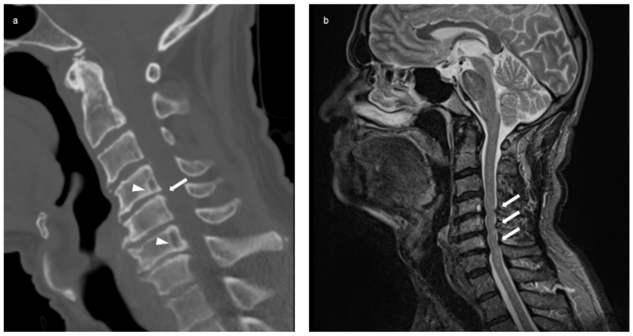

Figure **S2a:** Cervical spine CT demonstrates multilevel cervical spondylosis with osteophytosis of the vertebral bodies and irregularities of the vertebral endplates as well as cortical layer defects of the vertebral bodies and C3 transverse process *(white arrow*) A gaseous degeneration of the C5 and C6 vertebral bodies, reduction of the C3 - C6 vertebral heights and protrusions of the C3/C4 and C4/C5 intervertebral discs are also present *(white arrowheads).* These abnormalities cause severe multilevel spinal canal stenosis, yet no conflict with vertebral arteries is detected. Figure **S2b:** Cervical spine MRI demonstrates spondylosis with spinal canal stenosis, most severely at C3/C4, C4/C5 and C5/C6 levels and a contrast-enhancing inflammation and oedema areas at the mentioned levels (*white arrows).*
